# M. Van Acker: From flux to frame. Designing infrastructure and shaping urbanization in Belgium: Leuven University Press, Leuven, 2014, p. 464, 9789058679581

**DOI:** 10.1007/s10901-016-9501-x

**Published:** 2016-02-20

**Authors:** J. Rouwendal

**Affiliations:** 1 VU University Amsterdam, Amsterdam, The Netherlands; 2 Tinbergen Institute, Amsterdam, The Netherlands; 3 NAKE, Amsterdam, The Netherlands

This book, a re-edited version of the author’s PhD thesis defended at the University of Leuven, describes the mutual relationship between infrastructure development and urbanization on the basis of three Belgian case studies. The first and longest of these refers to the Campine, the second to the coast line and the third to greater Antwerp. The ‘colonization’ of the Campine area, not much more than a wilderness in the early nineteenth century, was realized in close intertwinement of the construction of canals and the location of industry. The urbanization of the Belgian coastline was unthinkable without the appropriate traffic infrastructure that enabled visitors to reach the sea and travel along the coast. And a city like Antwerp cannot be imagined without appropriate railways and motorways.

The author describes the history of the development of large infrastructure projects in connection with urbanization. In the case of the Campine, the story he tells suggests that the infrastructural development was in the lead position. Without the canals, early industrialization would have been impossible. In case of the coast, the reader gets the idea that decision making with respect to infrastructure was a deliberate reaction to the known presence of a potentially large demand for trips to the shore among the increasingly wealthy Belgian consumers who were eager to benefit from this phenomenon. The history of infrastructure in and around Antwerp is less clear.

It is a commonplace to state that urban areas need infrastructure. However, the author’s research shows at several places that the development of traffic infrastructure is not just a response to growing needs for transport of people and commodities but has itself a structuring effect on the area concerned and helps determine its future. City development is not just expansion of an urban area through the construction of more residential and industrial areas which should be appropriately facilitated by infrastructure. It is also the case that the location of existing and planned infrastructure has an impact on the development and location of the houses, other buildings, recreational areas, et cetera that will be constructed. Moreover, in the course of time the significance and meaning of the infrastructure will change and this may open possibilities for new developments, while prohibiting others. It is the description of this mutual relationship that makes the book interesting and valuable reading.

The book is by and large descriptive. It relates the developments and puts them in appropriate contexts, but the analysis does not lead to clear recommendations for infrastructure or urbanization policy, although—as said—some of the stories are highly suggestive in this respect. Contrary to what the author appears to think, urban economic analysis has paid some attention to this topic. The main contribution to this branch of the literature is probably Baum-Snow’s (2007) analysis of the impact of highways on suburbanization in U.S. metropolitan areas, which he showed to be substantial. This result, which established that highway construction did not merely follow urban development but was itself an important cause of it, seems to fit well with the analysis of this book.
